# Nurse Staffing Calculation in the Emergency Department - Performance-Oriented Calculation Based on the Manchester Triage System at the University Hospital Bonn

**DOI:** 10.1371/journal.pone.0154344

**Published:** 2016-05-03

**Authors:** Ingo Gräff, Bernd Goldschmidt, Procula Glien, Sophia Klockner, Felix Erdfelder, Jennifer Lynn Schiefer, Daniel Grigutsch

**Affiliations:** 1 Emergency Department, University Hospital Bonn, Bonn, Germany; 2 Department of Anesthesiology, University Hospital Bonn, Bonn, Germany; 3 Process Management, University Hospital Bonn, Bonn, Germany; 4 Department for Plastic, Hand, Reconstructive and Burn Surgery, Hospital Köln-Merheim, Köln, Germany; 5 Department of Anesthesiology, Hospital Mechernich, Mechernich, Germany; Yokohama City University, JAPAN

## Abstract

**Background:**

To date, there are no valid statistics regarding the number of full time staff necessary for nursing care in emergency departments in Europe.

**Material and Methods:**

Staff requirement calculations were performed using state-of-the art procedures which take both fluctuating patient volume and individual staff shortfall rates into consideration. In a longitudinal observational study, the average nursing staff engagement time per patient was assessed for 503 patients. For this purpose, a full-time staffing calculation was estimated based on the five priority levels of the Manchester Triage System (MTS), taking into account specific workload fluctuations (50th-95th percentiles).

**Results:**

Patients classified to the MTS category red (n = 35) required the most engagement time with an average of 97.93 min per patient. On weighted average, for orange MTS category patients (n = 118), nursing staff were required for 85.07 min, for patients in the yellow MTS category (n = 181), 40.95 min, while the two MTS categories with the least acute patients, green (n = 129) and blue (n = 40) required 23.18 min and 14.99 min engagement time per patient, respectively. Individual staff shortfall due to sick days and vacation time was 20.87% of the total working hours. When extrapolating this to 21,899 (2010) emergency patients, 67–123 emergency patients (50–95% percentile) per month can be seen by one nurse. The calculated full time staffing requirement depending on the percentiles was 14.8 to 27.1.

**Conclusion:**

Performance-oriented staff planning offers an objective instrument for calculation of the full-time nursing staff required in emergency departments.

## Introduction

### Background

Centralization of processes for intra-hospital emergency treatment through the establishment of central emergency departments (ED) in Europe is supposed to improve the quality of nursing as well as reduce medical engineering and equipment expenses, especially through a more efficient nursing workforce [1]. Labor costs account for 60–70% of the overall expenses in a central emergency department (of which 30% are for nursing alone) [2].

### Meaning of the study

The traditional calculation of full-time staff based on the predicted number of emergency patients per year is not sufficiently sensitive for the calculation of nursing staff in an emergency department [3], as nursing minutes per patient may represent only a preliminary value [4]. Organizational structures and procedures such as the emergency classification at the start of the process in emergency care ought to be taken into account. The LEP method of recording and processing documentation in health care (LEP) for the quantification of nursing care efforts, as applied in Switzerland in emergency rooms in the initial stages, does not yet exist in Germany [5]. Since the emergency department is not included in the Diagnoses Related Groups (DRG)–i.e. with its own cost calculation—cost-oriented calculation cannot be used for human resource planning for patients who are hospitalized via an emergency department [6].

### Aim of the study

The aim of the present study was to verify calculations of required nursing staff in the emergency department (ED) of a clinic providing maximum care, based on a structured recording of activity. For the first time, the patient-specific engagement time in emergency care was prospectively measured for the five priority levels of the Manchester Triage System (MTS). Staff engagement that were not directly related to patient care were recorded separately. Individual staff shortfalls and variations in the patient flow at the ED were also ascertained and carefully taken into account in the calculation of necessary full time staff.

## Materials and Methods

### Main features of emergency care at the University Hospital Bonn

A detailed listing of patient numbers, including triage level, the spatial structures and the organizational structures, etc., are given in the supporting information (S1 Table).

### Sampling

In a longitudinal observational study, the specific patient engagement time of all nurses during emergency care in the ED was assessed. The observation period spanned from October 2010 to June 2011; and sampling was conducted for 24 hours periods on randomly selected days. During this period, patient engagement times were measured by external observers for a total of 503 emergency patients. The number of medical and nursing employees as well as the skill level remained steady during the observation period

### Measuring methods / data collection

#### Patient-related engagement times

The observations and, thus, measurement of the engagement time pertained to direct patient-interactions. Observers followed the patients from time of arrival until discharge; patient-related activities prior to arrival and after leaving the ED were likewise recorded. For documentation of engagement time, five aspects were defined as performance variables: set-up, initial assessment, emergency care, monitoring time in the holding area, and other activities (S2 Table).

#### Patient-independent engagement times

Staff engagement times that were not directly related to patient care, but which are indispensable prerequisites for proper care were recorded separately. These were essentially related to logistic and procurement activities, such as the checking of all emergency equipment (backpacks, ambulances, etc.) in the ED. This is done according to a specified algorithm with the use of checklists by the respective nurses. In addition, purchases, such as the ordering and storing of pharmaceuticals, placing of external orders, sterile equipment logistics and equipment maintenance were recorded.

### Data analysis / statistics

The calculation of the number of necessary full time staff was carried out using the available "open source" software "R" (Version 3.1.2 / http://www.r-project.com/). Confidence intervals were evaluated by non-parametric bootstrapping (10000 bootstrap samples per confidence interval) using SPSS software version 23 (Chicago/IL, USA).

### Ethics Statement / Privacy Policy

The chairman of the local ethics committee (Prof. Dr. K. Racké, University of Bonn) declared after the presentation of the study design, that the data collection did not constitute a research project. Therefore a consultation by the ethics committee, in accordance with the code of medical ethics §15 (http://www.aekno.de/page.asp?pageID=57#_15) of the General Medical Council, was not required. The patients’ consent for this survey was given by the general terms and conditions of the University of Bonn (AVB § 16 para. 3). The Declaration of Helsinki was observed.

Before carrying out the study, written, informed consent of the responsible ethics committee, the Staff Council and the Head of Nursing were obtained. It was determined that written consent of the individual nurses was not necessary. Verbal informing of the nurses prior to the study was considered sufficient. Therefore all the nurses were informed about the details of the data collection in an official team meeting before study commencement.

### Engagement times

In order to present the different durations of involvement (Nurse Engagement Time NET) of each participating discipline as accurately as possible, the times determined for each MTS-Level (Table 1A) are calculated and weighted separately for the discipline’s real patient volumes.

**Table 1:** Nurse engagement times.

**a) Patient-related engagement times according to MTS categories**^**a**^					
	MTS 1	MTS 2	MTS 3	MTS 4	MTS 5
number (n)	35	118	181	129	40
set-up time	29.6	8.13	2.29	1.36	1.13
first assessment	9.15	9.43	9.53	8.72	8.38
emergency care	26.58	16.83	10.37	5.6	2.79
holding area	22.74	42.55	14.05	4.28	0
miscellaneous	9.86	8.13	4.72	3.22	2.69
**total**	**97.93**	**85.07**	**40.95**	**23.18**	**14.99**
**b) Patient-independent engagement times**^**b**^					
	logistics	procurement	other activities		total
engagement time	760	200	111		1071

^a^ average engagement times in each category and as total engagement time; shown in minutes.

^b^ average engagement times without assigned MTS category; shown as hours per year.

$$\begin{array}{l} {Nurse\;Engagement\;Time}_{MTS}\\ \;={average\;treatment\;time}_{Discipline\;a}\\ \;\times \;{share\;of\;patient\;volume}_{Discipline\;a}+\cdots \\ \;+\;{average\;treatment\;time}_{Discipline\;n}\\ \;\times \;{share\;of\;patient\;volume}_{Discipline\;n} \end{array}$$

In the supporting information (S1 Fig) the actual distribution of engagement time is shown using a Box Plot.

Since the identified patient-independent engagement times during the observation period cannot be merely attributed to the determined MTS categories, engagement times are given as full-time staff in annual hours. The largest share of patient-independent engagement time was spent on logistical activities (e.g., checking of emergency equipment). The second largest portion was used for procurement activities (Table 1B).

### Calculation of required staff

#### Calculation of patient care

First, data from the actual patient collective from the year 2010 with a total of 21,899 emergency patients were grouped by day of the week and hour of the day, resulting in a total of 168 tables that depict the volume of patients according to the five MTS categories for each hour of each day.

As an example, the record for Monday, 00:00–00:59 hours, is shown in the supporting information (S3 Table).

By multiplying the number of patient arrivals within one hour with the identified engagement times corresponding to MTS category on the sample day, the overall engagement time can be calculated thus:

$${NET(total)}_{d,t}={n(red)}_{d,t}\times {NET(red)}_{d,t}+{n(orange)}_{d,t}\times {NET(orange)}_{d,t}+{n(yellow)}_{d,t}\times {NET(yellow)}_{d,t}+{n(green)}_{d,t}\times {NET(green)}_{d,t}+{n(blue)}_{d,t}\times {NET(blue)}_{d,t}$$

NET = nurse engagement time

n = number of patients

d = date

t = time

Thus, the work force required to adequately handle the number of expected patients was be determined for each hour of the day. Obviously, due to fluctuations in the number of incoming emergency patients, this cannot be a daily constant and the workload differs accordingly.

In order to understand the fluctuating workload in mathematical terms, a corresponding empirical distribution is presented with the data of the actual collective in a mathematical function (S2 Fig): for example, in order to have sufficient staff for 85% of cases on a Monday between 0:00 and 0:59 hours (85th percentile), i.e., allowing for fluctuations in patient flow, 126 min of staff time must be made available.

Applying this method to each hour of each day results in 7 (d) * 24 (h) = 168 value tables and from each value table, the specific empirical distribution function and the corresponding percentiles can be determined. The Bootstrapping method was used to determine confidence intervals, given in the supporting information (S4 Table). However, in most cases the thus calculated staff requirements are not an integer and, since nurses are not divisible, the staff requirement must be rounded up. In addition, a certain minimum staffing level is necessary for running a large emergency department even at times of slow patient flow. Hence, if, for example, only about 100 minutes of the hospital’s work force need to be available, this is always calculated to be least two nurses.

Based on the calculated values, it was possible to calculate tailored staffing levels for every day of the week according to time of day:

$${staff\;hours}_{\text{wd},\text{t}}=2\geq \;\left\lceil {percentile}_{x}\left({NET(total)}_{d_{a},t}+\ldots +{NET(total)}_{d_{b},t}\right)\right\rceil$$

x = selected coverage level (percentile)

wd = week day

d = date

t = time

a = starting date

b = end date

The number of staff hours that are required to cover the selected period are calculated by multiplying the value obtained with the number of occurrences of the corresponding day in the period *a* to *b* (here: one year):

$${annual\;staff\;hours}_{wd,t}={staff\;hours\;}_{wd,t}\times n_{wd\left(a\ldots b\right)}mit$$

wd = week day

t = time

n = number of days of the week for the period a to b

a = starting date

b = end date

**Fig 1:**
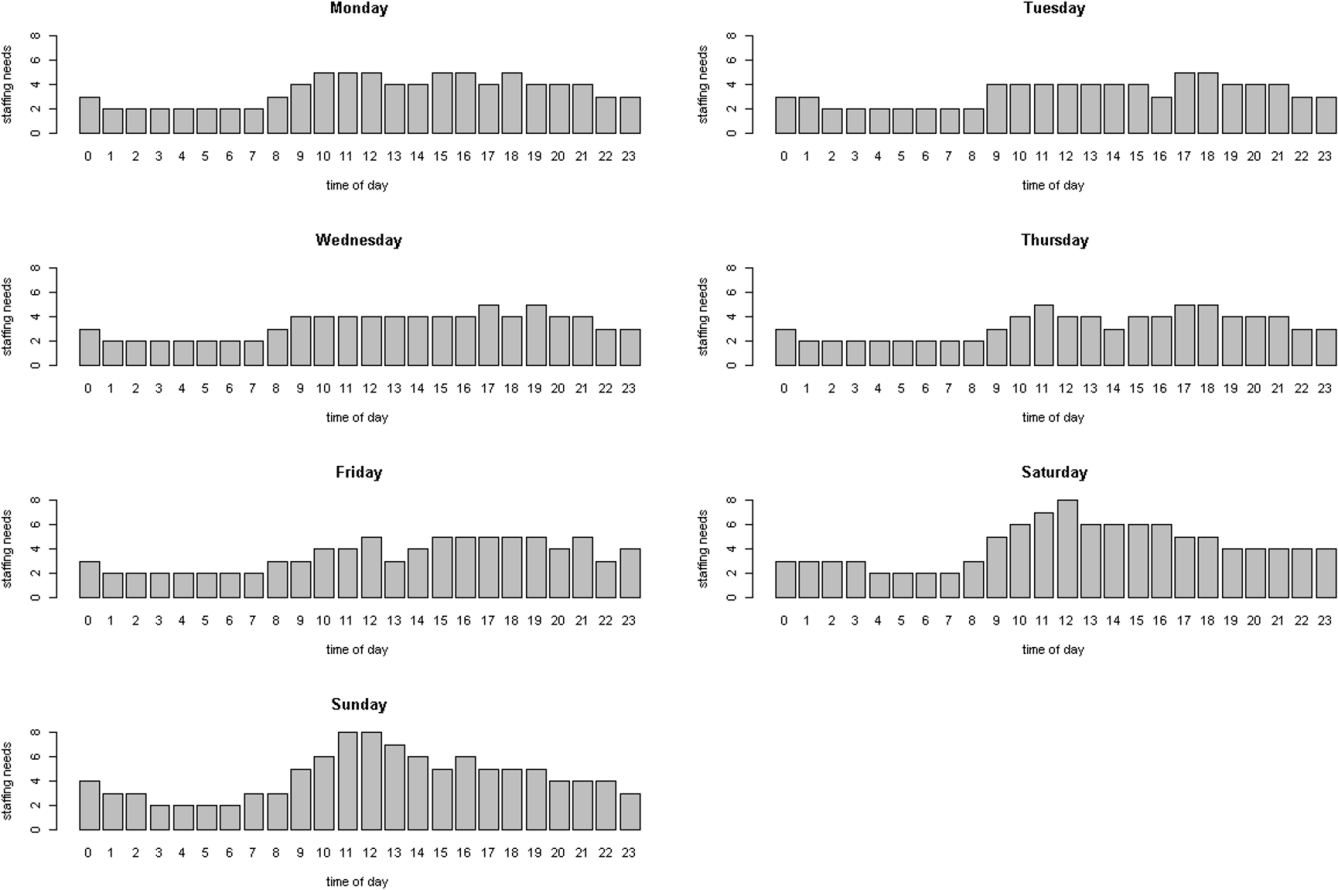
Staffing levels. The figure shows the minimum staffing levels on the each day of the week to adequately treat incoming patients in at least 85% of the observed hours.

Fig 1 shows the minimum staffing levels on each day of the week in order to adequately engage with all incoming patients at least 85% of the time.

Adding the calculated staffing requirements for each hour and each day of the week lead to the year overall staff hours necessary for adequate patient care for the total cohort of 2010:

$${annual\;staff\;hours}_{total}=\sum_{wd,t=Monday,0\;a.m.}^{Sunday,11\;p.m.}{\text{annual staff hours}}_{\text{wd},\text{t}}$$

wd = week day

t = time

#### Conversion of numbers to full time nurses

With 254 annual working days for 2010 in the federal state of North Rhine-Westphalia, and a 38.5-hour week, this results in an annual total of 1,955 working hours at the University Hospital Bonn.

For the calculation of needed full time nurses in this study, the actual shortfall rate in the ED was detected to be 20.87% (S5 Table) resulting in a net working time at the ED of 1,547 hours per year. When the resulting total staff hours were divided by the 1,547 hours net working time, the number of necessary full time staff for direct patient care could be calculated for each chosen percentile.

During the observation period, patient-independent engagement time (Table 1B) was on average 1,071 hours per year.

In relation to the net annual working time of a full-time nurse (1,071:1,547), this results in 0.7 full-time nurses. The necessary total number of full-time nurses could therefore be calculated by adding percentile-dependent working hours to the determined number of nurses needed for patient-independent engagement times (Table 2).

**Table 2:** Calculated number of full-time staff^*^.

percentile	50	55	60	65	70	75	80	85	90	95
working hours	21,844	22,627	23,982	25,180	26,172	27,578	29,403	31,957	34,879	40,878
patient-related full-time staff	14.12	14.63	15.5	16.28	16.88	17.83	19.01	20.66	22.55	26.42
patient-independent full-time staff	0.7	0.7	0.7	0.7	0.7	0.7	0.7	0.7	0.7	0.7
**full-time staff total**	**14.82**	**15.33**	**16.2**	**16.98**	**17.58**	**18.53**	**19.71**	**21.36**	**23.25**	**27.12**

*The total number of full-time staff for the calculated percentile-related working hours of patient engagement time and patient-independent engagement time.

#### Additional required staff

The ED at the University Hospital Bonn employs a coordinating nurse, who is exclusively responsible for the coordination of emergency care. Depending on the working hours of the coordinating nurse, additional human resources are required as an emergency department of this size needs a coordinating senior nurse at all times. In addition to organizing daily business, such as setting up the duty roster or staff appraisals, aspects of quality and risk management (certifications / audits) now require considerable time and can no longer be done on the side.

## Results

The MTS category red (n = 35) showed the longest patient engagement time with a weighted average of 97.93 minutes per patient. Of note, the "red" patients required a proportionately longer set-up time and a longer time of nursing emergency care. Patient engagement in the MTS category orange (n = 118) was on weighted average 85.07 minutes, while in the MTS category yellow (n = 181), it was 40.95 min.

The highest surveillance time in the holding area was found for the MTS category orange. In the two lowest MTS categories, shorter average engagement times were measured, with 23.18 min for the green MTS category (n = 129) and 14.99 min for the blue MTS level (n = 40).

The resulting total calculated working hours derived from this and based on patient volume, which are necessary for the running of the emergency department, are dependent on the percentile. These lie between 21,844 hours at the 50^th^ percentile and 40,878 hours if the 95^th^ percentile is selected. In the example above (85th percentile), this yielded a total of 31,957 working hours (Table 2).

Converted into number of full-time staff, this corresponds to between 14.82 to 27.12 full-time employees. For the 85th percentile, based on this example, this is 21.36 of full-time staff.

## Discussion

For the first time, nursing services providing emergency care and differentiated by MTS category were measured prospectively in an ED at a university hospital. Staff requirement calculations were performed using state-of-the art procedures, which take both fluctuating patient volume and individual staff shortfall rates into consideration.

Several studies have shown a direct correlation between the engagement of nursing staff in an emergency room and patient outcome [3,7]. Needleman et al. were able to demonstrate a clear correlation between number of nurses and prevention of sepsis, cardiac arrest, and pneumonia [8,9]. On the other hand, central emergency rooms are under tremendous economic pressure. Here, especially the under-financing in ambulatory emergency care should be pointed out (http://www.dgina.de./media/veroeffent/20090729_Positionspapier_2009_07_08_MB_F.pdf).

Competent management is essential in order to maintain the balance between the necessary efficiency on the one hand and required quality and risk management on the other hand.

For example, a work load profile to determine the maximum patient volume and an "anticipatory" roster are essential for every ED. Classic three-shift models are not suitable for EDs and must be replaced with roster models with overlapping periods of service. Nevertheless, peak occurrences, whereby the patient load significantly exceeds the processing capacity, will remain unpredictable for every emergency room across the world.

As a possible problem-solving approach, the present study offers a staggered calculation based on statistics, which not only considers the average volume of patients, but also includes the fluctuations in patient volume for each hour of the day. With the exception of the patient engagement time, which presumably differs between emergency departments, it can be used in the same way for other hospitals. Taking into account economic aspects at an equally high level of quality of care, Gries et al. believe that in the medical field, a nursing team that ensures adequate patient care at peak times in 85% of cases is sufficient [10].

Considering existing medical and economic conditions, the necessary staff can be calculated so that patients are attended to optimally while at the same time, contingency of nursing staff during times of low patient flow is reduced as much as possible [11,12]. However, further steps are required. In the ED at the University Hospital Bonn, for example, the coordinating nurse is of great importance, especially during periods of maximum patient flow, during which he or she monitors several parallel processes and, thus, steers the ED operations.

For an exceptional situation, such as the simultaneous arrival of multiple emergency room patients, the additional consultation of a Medical Emergency Team would be conceivable in order to maintain the capacity of the emergency department [13].

### Key figures in comparison

The German hospital institute (Deutsches Krankenhausinstitut, DKI) has published key figures for clinics with decentralized outpatient departments. These, however, differ considerably depending on the department. Thus, values of 18 to 75 minutes are stated per patient [4]. Considering the number of cases that can be attended by one nurse in one year, numbers vary between 1,360–4,080 cases.

Calculations including the so-called normative approximate value show that a nurse in a central emergency room with 20,000–25,000 patients per year should be able to attend to 110 to 120 patients a month. In addition, the average case severity of the emergency patient involving the Case Mix Index (CMI) is taken into account in the calculation. A factor for special patient groups, such as patients with multiple trauma (+ 0.2 full-time nurses) has been added. For the ED of the University Hospital Bonn, with an average CMI of emergency patients of 1.42 and 21,899 patients, this would result in 21.7 full time nurses [14].

In 2010, a survey performed by the German society of interdisciplinary emergency and acute medicine (Deutsche Gesellschaft Interdisziplinäre Notfall- und Akutmedizin, DGINA) revealed that in the emergency departments of 22 clinics, an average of 128 patients (median 112) are attended by one nurse per month [15].

In a US emergency department (Long Island, NY) Fullam et al. used the engagement times of a five-level triage system to calculate the number of full time staff. With an underlying net amount of working time of 1,533 hours per year (22% downtime) a full time staff of 30.64 nurses was needed for 30,397 emergency patients per year without administration. Extrapolated to the 21,899 patients in our ED, this corresponds to 22 full time nurses [16]. In a similar study from a New Zealand emergency department with 65,000 emergency patients per year, the so-called "direct nursing care time" was evaluated within the five-stage Australasian triage system. There, the calculated average engagement time was with 49 min per case close to the engagement time of the present study [17]. Table 3 shows the various methods to calculate the number of staff needed in comparison.

**Table 3:** Required for full-time staff depending on different methods of investigation.

study	patients per month per nursing staff	triage-system	staff default time (%)
DKI ^a^	113–340	not provided	20%
Schachinger et al.^b^ [14]	77–84	mainly established	not specified
DGINA ^c^	128 (mean)	partially	not specified
	112 (median)		
Fullam et al.[16]	83	established	22%
UKB ^d^	123 (50th percentile)	established	21%
	119 (55th percentile)		
	113 (60th percentile)		
	107 (65th percentile)		
	104 (70th percentile)		
	98 (75th percentile)		
	93 (80th percentile)		
	85 (85th percentile)		
	78 (90th percentile)		
	67 (95th percentile)		

^a^ DKI = German Hospital Institute; normative approximation is shown in more detail in the text (Case Mix Index CMI = 1,42)

^b^ Normative approximate value x CMI + Factor Polytrauma (20–25,000 Pat. / Year)

^c^ DGINA = German society of interdisciplinary emergency and acute medicine (member survey)

^d^ UKB = University Hospital of Bonn / Numbers are staggered by percentile (Table 2)

## Limitations

When process times are surveyed by external observers, the so-called "Hawthorne effect" has been described [18], i.e. the presence of external observers itself could have led to changes in the process flow. We consider this effect to be negligible due to the standardized ED courses of action. The engagement times identified in the present study are based on the activities and do not evaluate the nursing staff in terms of effectiveness of the measures applied. Previously published data, however, show that the ED at the University of Bonn has undergone a process of workflow optimization with an increase of efficiency in the year 2009 [1].

The collected data do not exactly reveal when patients required engagement time during their stay. Based on experience, it may be assumed that the majority of direct patient engagement was at the beginning of a patient’s stay due to the requirement of immediate triage on arrival of an emergency patient. Therefore, the entire engagement time took place within the first hour of a patient’s stay in the emergency department.

In addition, minimum time periods of work shifts are not taken into account. Thus, peak load covers, where an additional nurse would be required, are not allowed for an hour or two as stipulated by the German Working Hours Act.

## Conclusion

The presented methodology, based on the MTS, enables timely adjustments to changing requirements due to alterations in patient flow in the daily practice of an emergency department. Changes in patient collectives, working conditions and quality requirements require periodic re-evaluations in staff requirement calculation. Particular focus must be kept on future demographic developments. Already, professional societies call for special assessment of geriatric patients in emergency departments[19].

Adequate calculations of the required full time staff in emergency departments represent a particular challenge. Existing computational methods cannot be used readily as the gold standard. The efficient use of personnel is confronted with several quality and risk management matters that have to be rectified simultaneously. Nevertheless, the number of nurses does affect patient outcome. Pertinent to discussions about the quality of emergency care and increasing economic pressures, performance-oriented staff planning based on the five stages of the Manchester Triage System provides an objective instrument for the calculation of the nursing staff and is a valuable contribution for the planning of human resources in emergency departments.

## Supporting Information

S1 Fig Distribution of direct engagement time. (DOCX)

S2 Fig Empirical distribution function. Exemplary illustration of an empirical distribution function for the period on Mondays between12:00 a.m. - 12:59 a.m. Accordingly, in order to, for example, have sufficient staff in 85% of cases in this period (85% percentile); it is necessary to have 126 minutes of staff time available.
(TIFF)

S1 Table Main features of emergency care at the University Hospital Bonn. (DOCX)

S2 Table Definition of the performance variables. (DOCX)

S3 Table Number of patient arrivals (exemplary record). An example of a record for Monday 12:00 a.m. - 12:59 a.m. with the number of patients in the respective MTS categories.
(DOCX)

S4 Table Confidence intervals. Confidence intervals were evaluated by non-parametric bootstrapping (10000 bootstrap samples per confidence interval).
(DOCX)

S5 Table Staff shortfall times in the interdisciplinary emergency department*. (DOCX)
