# Supplementary figures and images for: Nurse Staffing Calculation in the Emergency Department - Performance-Oriented Calculation Based on the Manchester Triage System at the University Hospital Bonn
Source: PLoS One. 2016 May 3;11(5):e0154344. doi: 10.1371/journal.pone.0154344 (PMC4854466; doi:10.1371/journal.pone.0154344)

**S1 Figure. Distribution of engagement times**


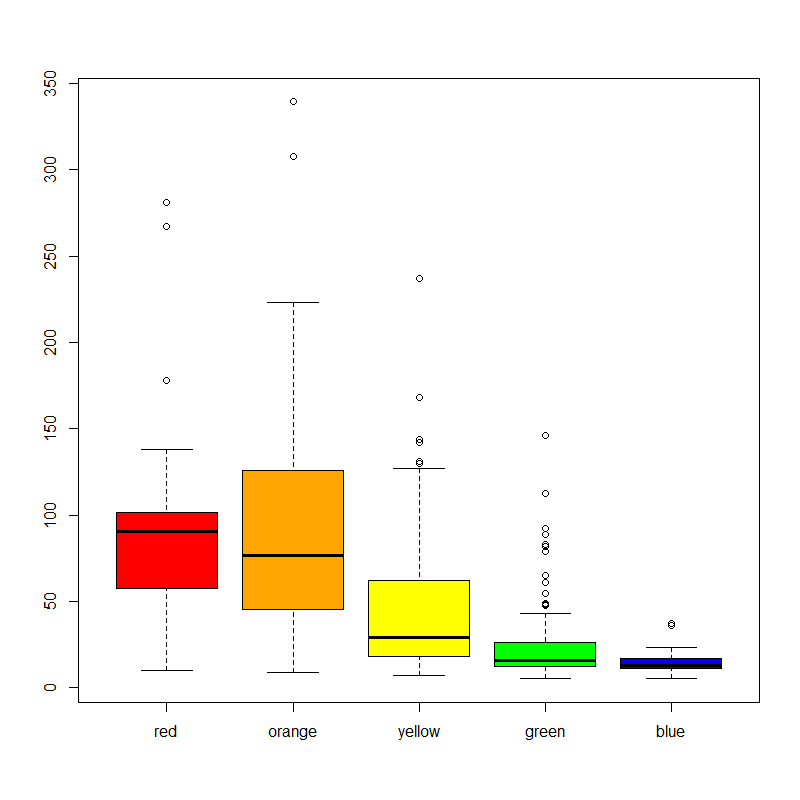

Supplement: S1 Fig — (DOCX) [file pone.0154344.s001.docx]

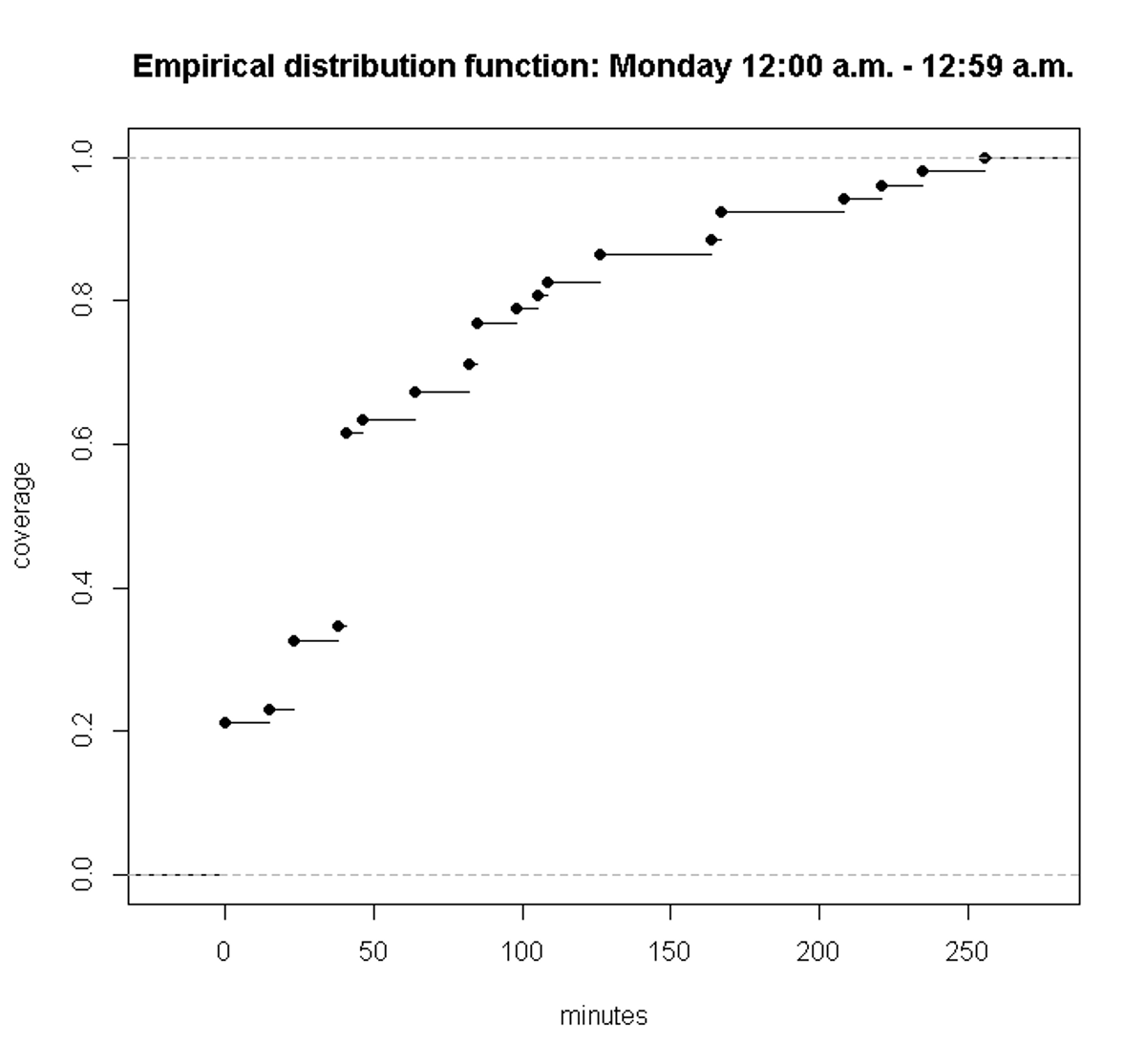

Supplement: S2 Fig — Exemplary illustration of an empirical distribution function for the period on Mondays between12:00 a.m. - 12:59 a.m. Accordingly, in order to, for example, have sufficient staff in 85% of cases in this period (85% percentile); it is necessary to have 126 minutes of staff time available. (TIFF) [file pone.0154344.s002.tiff]
