# Supplementary material for: Nurse Staffing Calculation in the Emergency Department - Performance-Oriented Calculation Based on the Manchester Triage System at the University Hospital Bonn
Source: PLoS One. 2016 May 3;11(5):e0154344. doi: 10.1371/journal.pone.0154344 (PMC4854466; doi:10.1371/journal.pone.0154344)
Supplement: S1 Table — (DOCX) [file pone.0154344.s003.docx]

## S1 Table. Main features of emergency care at the University Hospital Bonn

| - MTS levels: 1.7% red, 18.3% orange, 47.1% yellow, 28.8 % green, 4.1% blue |
| --- |
| - 11 departments |
| - 3 trauma rooms and 9 treatment rooms |
| - Holding area with five monitor places |
| - Primary care for all patients in the emergency department (no bypassing from preclinical to, e.g., ICU) |
| - Emergency treatment only, no elective or scheduled treatment of patients |
| - 600 emergency room patients per year |
| - Multi-regional trauma center |
| - Primary nursing concept at the beginning of all processes (administration, triage and department assignment by nurses) |
| - Employment of coordinating nurse |
| - Data administration by medical transcriptionists |
| - Patient transport through a transport service |
| - Combination of skills: nurses, specialist nurses and paramedics |
| - Average length of patient stay: 02:18 hrs. |
