# Supplementary material for: Nurse Staffing Calculation in the Emergency Department - Performance-Oriented Calculation Based on the Manchester Triage System at the University Hospital Bonn
Source: PLoS One. 2016 May 3;11(5):e0154344. doi: 10.1371/journal.pone.0154344 (PMC4854466; doi:10.1371/journal.pone.0154344)
Supplement: S2 Table — (DOCX) [file pone.0154344.s004.docx]

**S2 Table. Definition of the performance variables**

| Variables: |
| --- |
| Set-up time: engagement time that starts with preparation of the treatment room for the patient. This mainly includes preparation of the following: medical equipment, invasive procedures, medication, blood sampling and documentation. Waiting for the arrival of the patient as well as the restoring of the treatment room after the emergency were also included. |
| Initial assessment: time spent on basic administration, gathering of medical history including triage and collection of vital signs. |
| Emergency care: therapy, blood sampling, ECG, urine status, wound care, medical assistance, patient care, point of care testing, patient monitoring, measures for infection control etc. |
| Holding area: patient care in the monitoring area, which is equipped with a total of five patient monitors. Here, patients are waiting for further diagnostic or laboratory results. |
| Other activities: documentation, activity recording, communication with next of kin etc. |
