# Supplementary material for: Nurse Staffing Calculation in the Emergency Department - Performance-Oriented Calculation Based on the Manchester Triage System at the University Hospital Bonn
Source: PLoS One. 2016 May 3;11(5):e0154344. doi: 10.1371/journal.pone.0154344 (PMC4854466; doi:10.1371/journal.pone.0154344)
Supplement: S3 Table — An example of a record for Monday 12:00 a.m. - 12:59 a.m. with the number of patients in the respective MTS categories. (DOCX) [file pone.0154344.s005.docx]

**S3 Table. Number of patients arrival (exemplary record)**

An example of a record for Monday 12:00 a.m. - 12:59 a.m. with the number of patients in the respective MTS categories

| Date | n(red) | n(orange) | n(yellow) | n(green) | n(blue) |
| --- | --- | --- | --- | --- | --- |
| 01/04/2010 | 0 | 1 | 1 | 0 | 0 |
| 01/11/2010 | 0 | 1 | 0 | 1 | 0 |
| 01/18/2010 | 0 | 0 | 0 | 0 | 0 |
| 01/25/2010 | 0 | 0 | 0 | 0 | 0 |
| 02/01/2010 | 0 | 0 | 0 | 1 | 0 |
| 02/08/2010 | 0 | 3 | 0 | 0 | 0 |
| 02/15/2010 | 0 | 0 | 1 | 0 | 0 |
| 02/22/2010 | 0 | 0 | 1 | 0 | 0 |
| 03/01/2010 | 0 | 0 | 1 | 0 | 0 |
| 03/08/2010 | 0 | 0 | 1 | 0 | 0 |
| 03/15/2010 | 0 | 0 | 0 | 0 | 0 |
| 03/22/2010 | 0 | 0 | 0 | 0 | 0 |
| 03/29/2010 | 0 | 0 | 1 | 0 | 0 |
| 04/05/2010 | 0 | 1 | 2 | 0 | 0 |
| 04/12/2010 | 0 | 1 | 3 | 0 | 0 |
| 04/19/2010 | 0 | 1 | 0 | 0 | 0 |
| 04/26/2010 | 0 | 0 | 0 | 2 | 0 |
| 05/03/2010 | 0 | 0 | 2 | 0 | 0 |
| 05/10/2010 | 0 | 0 | 0 | 1 | 0 |
| 05/17/2010 | 0 | 0 | 0 | 1 | 0 |
| 05/24/2010 | 0 | 2 | 1 | 1 | 0 |
| 05/31/2010 | 0 | 0 | 0 | 0 | 1 |
| 06/07/2010 | 0 | 0 | 1 | 0 | 0 |
| 06/14/2010 | 0 | 0 | 0 | 0 | 0 |
| 06/21/2010 | 0 | 1 | 2 | 0 | 0 |
| 06/28/2010 | 0 | 0 | 0 | 0 | 0 |
| 07/05/2010 | 1 | 0 | 0 | 0 | 0 |
| 07/12/2010 | 1 | 0 | 3 | 0 | 0 |
| 07/19/2010 | 0 | 0 | 1 | 0 | 0 |
| 07/26/2010 | 0 | 1 | 0 | 0 | 0 |
| 08/02/2010 | 0 | 0 | 2 | 0 | 0 |
| 08/09/2010 | 0 | 0 | 1 | 0 | 0 |
| 08/16/2010 | 0 | 0 | 1 | 0 | 0 |
| 08/23/2010 | 0 | 0 | 1 | 0 | 0 |
| 08/30/2010 | 0 | 0 | 0 | 1 | 0 |
| 09/06/2010 | 0 | 0 | 0 | 0 | 0 |
| 09/13/2010 | 0 | 1 | 1 | 0 | 0 |
| 09/20/2010 | 0 | 0 | 4 | 0 | 0 |
| 09/27/2010 | 0 | 0 | 1 | 0 | 0 |
| 10/04/2010 | 0 | 0 | 0 | 0 | 0 |
| 10/11/2010 | 0 | 0 | 1 | 0 | 0 |
| 10/18/2010 | 0 | 0 | 0 | 0 | 0 |
| 10/25/2010 | 0 | 0 | 1 | 1 | 0 |
| 11/01/2010 | 0 | 0 | 1 | 0 | 0 |
| 11/08/2010 | 0 | 0 | 0 | 1 | 0 |
| 11/15/2010 | 0 | 0 | 1 | 0 | 0 |
| 11/22/2010 | 0 | 0 | 0 | 0 | 0 |
| 11/29/2010 | 0 | 0 | 0 | 0 | 0 |
| 12/06/2010 | 0 | 0 | 1 | 1 | 0 |
| 12/13/2010 | 0 | 0 | 0 | 1 | 1 |
| 12/20/2010 | 0 | 1 | 0 | 0 | 0 |
| 12/27/2010 | 0 | 0 | 2 | 1 | 0 |
