# Supplementary material for: Nurse Staffing Calculation in the Emergency Department - Performance-Oriented Calculation Based on the Manchester Triage System at the University Hospital Bonn
Source: PLoS One. 2016 May 3;11(5):e0154344. doi: 10.1371/journal.pone.0154344 (PMC4854466; doi:10.1371/journal.pone.0154344)
Supplement: S4 Table — Confidence intervals were evaluated by non-parametric bootstrapping (10000 bootstrap samples per confidence interval). (DOCX) [file pone.0154344.s006.docx]

| **S4 Table. Median & Confidence Intervals** | | | |  |  |  |  |  |  |  |  |  |
| --- | --- | --- | --- | --- | --- | --- | --- | --- | --- | --- | --- | --- |
| **Monday** |  |  |  |  |  |  |  |  |  |  |  |  |
| **Time (AM)** | **0:00 - 0:59h** | **1:00 - 1:59h** | **2:00 - 2:59h** | **3:00 - 3:59h** | **4:00 - 4:59h** | **5:00 - 5:59h** | **6:00 - 6:59h** | **7:00 - 7:59h** | **8:00 - 8:59h** | **9:00 - 9:59h** | **10:00 - 10:59h** | **11:00 - 11:59h** |
| lower 0.95 CI | 40.95 | 0.00 | 0.00 | 0.00 | 0.00 | 0.00 | 0.00 | 0.00 | 40.95 | 52.54 | 87.31 | 107.785 |
| median | 40.95 | 32.065 | 40.95 | 40.95 | 32.065 | 11.59 | 0.00 | 11.59 | 85.07 | 85.07 | 126.02 | 129.845 |
| upper 0.95 CI | 52.54 | 40.95 | 64.13 | 73.015 | 40.95 | 40.95 | 40.95 | 40.95 | 108.25 | 124.435 | 149.2 | 190.15 |
| **Time (PM)** | **12:00 - 12:59h** | **1:00 - 1:59h** | **2:00 - 2:59h** | **3:00 - 3:59h** | **4:00 - 4:59h** | **5:00 - 5:59h** | **6:00 - 6:59h** | **7:00 - 7:59h** | **8:00 - 8:59h** | **9:00 - 9:59h** | **10:00 - 10:59h** | **11:00 - 11:59h** |
| lower 0.95 CI | 85.07 | 83.215 | 83.485 | 81.9 | 107.785 | 100.985 | 104.155 | 105.08 | 86.19 | 87.31 | 64.13 | 40.95 |
| median | 115.55 | 92.62 | 122.85 | 97.93 | 160.79 | 124.63 | 148.735 | 145.16 | 124.435 | 146.03 | 81.9 | 64.13 |
| upper 0.95 CI | 156.5 | 126.02 | 146.03 | 149.2 | 183.6 | 166.97 | 169.675 | 170.795 | 149.2 | 165.385 | 126.02 | 92.62 |
| **Thuesday** |  |  |  |  |  |  |  |  |  |  |  |  |
| **Time (AM)** | **0:00 - 0:59h** | **1:00 - 1:59h** | **2:00 - 2:59h** | **3:00 - 3:59h** | **4:00 - 4:59h** | **5:00 - 5:59h** | **6:00 - 6:59h** | **7:00 - 7:59h** | **8:00 - 8:59h** | **9:00 - 9:59h** | **10:00 - 10:59h** | **11:00 - 11:59h** |
| lower 0.95 CI | 40.95 | 11.59 | 23.18 | 0.00 | 0.00 | 0.00 | 0.00 | 0.00 | 40.95 | 96.89 | 91.5 | 95.075 |
| median | 64.13 | 40.95 | 40.95 | 0.00 | 0.00 | 0.00 | 0.00 | 11.59 | 60.035 | 108.25 | 106.665 | 122.85 |
| upper 0.95 CI | 83.485 | 64.13 | 81.9 | 32.07 | 40.95 | 0.00 | 40.95 | 40.95 | 73.015 | 126.02 | 124.435 | 126.02 |
| **Time (PM)** | **12:00 - 12:59h** | **1:00 - 1:59h** | **2:00 - 2:59h** | **3:00 - 3:59h** | **4:00 - 4:59h** | **5:00 - 5:59h** | **6:00 - 6:59h** | **7:00 - 7:59h** | **8:00 - 8:59h** | **9:00 - 9:59h** | **10:00 - 10:59h** | **11:00 - 11:59h** |
| lower 0.95 CI | 126.02 | 81.9 | 83.485 | 92.1 | 66.835 | 106.665 | 109.37 | 115.55 | 105.08 | 83.485 | 64.13 | 32.065 |
| median | 134.635 | 115.745 | 108.25 | 108.25 | 87.31 | 136.97 | 147.615 | 146.03 | 126.02 | 105.08 | 81.9 | 51.15 |
| upper 0.95 CI | 168.555 | 133.57 | 129.845 | 136.025 | 126.02 | 166.97 | 195.56 | 151.44 | 166.97 | 128.725 | 113.095 | 81.9 |

| **Wednesday** |  |  |  |  |  |  |  |  |  |  |  |  |
| --- | --- | --- | --- | --- | --- | --- | --- | --- | --- | --- | --- | --- |
| **Time (AM)** | **0:00 - 0:59h** | **1:00 - 1:59h** | **2:00 - 2:59h** | **3:00 - 3:59h** | **4:00 - 4:59h** | **5:00 - 5:59h** | **6:00 - 6:59h** | **7:00 - 7:59h** | **8:00 - 8:59h** | **9:00 - 9:59h** | **10:00 - 10:59h** | **11:00 - 11:59h** |
| lower 0.95 CI | 23.18 | 23.18 | 0.00 | 0.00 | 0.00 | 0.00 | 0.00 | 0.00 | 40.95 | 83.485 | 75.72 | 85.07 |
| median | 40.95 | 40.95 | 23.18 | 23.18 | 0.00 | 0.00 | 0.00 | 32.065 | 60.035 | 106.665 | 106.665 | 106.665 |
| upper 0.95 CI | 81.9 | 85.07 | 40.95 | 40.95 | 40.95 | 32.07 | 23.18 | 40.95 | 85.07 | 126.02 | 126.02 | 126.02 |
| **Time (PM)** | **12:00 - 12:59h** | **1:00 - 1:59h** | **2:00 - 2:59h** | **3:00 - 3:59h** | **4:00 - 4:59h** | **5:00 - 5:59h** | **6:00 - 6:59h** | **7:00 - 7:59h** | **8:00 - 8:59h** | **9:00 - 9:59h** | **10:00 - 10:59h** | **11:00 - 11:59h** |
| lower 0.95 CI | 81.9 | 83.485 | 92.1 | 106.665 | 136.275 | 130.54 | 105.08 | 126.02 | 105.08 | 81.9 | 66.835 | 40.95 |
| median | 105.08 | 108.25 | 128.725 | 141.435 | 166.97 | 154.61 | 126.02 | 147.615 | 117.135 | 99.595 | 83.485 | 64.13 |
| upper 0.95 CI | 126.02 | 147.615 | 168.09 | 166.97 | 190.15 | 190.15 | 165.58 | 179.875 | 138.73 | 126.02 | 110.585 | 108.25 |
| **Thursday** |  |  |  |  |  |  |  |  |  |  |  |  |
| **Time (AM)** | **0:00 - 0:59h** | **1:00 - 1:59h** | **2:00 - 2:59h** | **3:00 - 3:59h** | **4:00 - 4:59h** | **5:00 - 5:59h** | **6:00 - 6:59h** | **7:00 - 7:59h** | **8:00 - 8:59h** | **9:00 - 9:59h** | **10:00 - 10:59h** | **11:00 - 11:59h** |
| lower 0.95 CI | 40.95 | 23.18 | 0.00 | 0.00 | 0.00 | 0.00 | 0.00 | 0.00 | 19.085 | 66.835 | 73.015 | 92.62 |
| median | 55.245 | 40.95 | 0.00 | 32.065 | 40.95 | 20.475 | 11.59 | 23.18 | 40.95 | 85.07 | 106.665 | 126.02 |
| upper 0.95 CI | 85.07 | 81.9 | 32.07 | 40.95 | 46.36 | 40.95 | 40.95 | 40.95 | 52.54 | 122.85 | 126.02 | 159.67 |
| **Time (PM)** | **12:00 - 12:59h** | **1:00 - 1:59h** | **2:00 - 2:59h** | **3:00 - 3:59h** | **4:00 - 4:59h** | **5:00 - 5:59h** | **6:00 - 6:59h** | **7:00 - 7:59h** | **8:00 - 8:59h** | **9:00 - 9:59h** | **10:00 - 10:59h** | **11:00 - 11:59h** |
| lower 0.95 CI | 95.075 | 64.13 | 60.035 | 81.9 | 94.805 | 87.31 | 108.25 | 87.31 | 106.665 | 105.08 | 46.36 | 40.95 |
| median | 122.85 | 85.07 | 85.07 | 103.69 | 106.665 | 127.14 | 146.03 | 124.435 | 146.03 | 124.435 | 85.07 | 81.9 |
| upper 0.95 CI | 137.61 | 122.85 | 126.02 | 136.025 | 126.02 | 176.975 | 163.995 | 149.2 | 178.56 | 165.385 | 105.08 | 86.19 |

| **Friday** |  |  |  |  |  |  |  |  |  |  |  |  |
| --- | --- | --- | --- | --- | --- | --- | --- | --- | --- | --- | --- | --- |
| **Time (AM)** | **0:00 - 0:59h** | **1:00 - 1:59h** | **2:00 - 2:59h** | **3:00 - 3:59h** | **4:00 - 4:59h** | **5:00 - 5:59h** | **6:00 - 6:59h** | **7:00 - 7:59h** | **8:00 - 8:59h** | **9:00 - 9:59h** | **10:00 - 10:59h** | **11:00 - 11:59h** |
| lower 0.95 CI | 23.18 | 23.18 | 0.00 | 0.00 | 0.00 | 0.00 | 0.00 | 0.00 | 40.95 | 81.9 | 64.13 | 85.07 |
| median | 40.95 | 40.95 | 0.00 | 14.99 | 0.00 | 0.00 | 23.18 | 0.00 | 81.9 | 85.07 | 108.25 | 126.02 |
| upper 0.95 CI | 40.95 | 40.95 | 40.95 | 40.95 | 40.95 | 40.95 | 40.95 | 40.95 | 85.07 | 108.25 | 126.02 | 131.43 |
| Time (PM) | **12:00 - 12:59h** | **1:00 - 1:59h** | **2:00 - 2:59h** | **3:00 - 3:59h** | **4:00 - 4:59h** | **5:00 - 5:59h** | **6:00 - 6:59h** | **7:00 - 7:59h** | **8:00 - 8:59h** | **9:00 - 9:59h** | **10:00 - 10:59h** | **11:00 - 11:59h** |
| lower 0.95 CI | 64.13 | 87.31 | 97.93 | 126.02 | 151.44 | 128.26 | 146.03 | 105.08 | 108.25 | 87.31 | 87.31 | 64.13 |
| median | 108.25 | 108.25 | 126.02 | 164.19 | 172.38 | 163.8 | 186.98 | 146.03 | 131.43 | 144.29 | 108.25 | 85.07 |
| upper 0.95 CI | 126.02 | 128.26 | 149.2 | 190.15 | 207.38 | 186.98 | 195.56 | 172.38 | 172.38 | 163.8 | 126.02 | 126.02 |
| **Saturday** |  |  |  |  |  |  |  |  |  |  |  |  |
| Time (AM) | **0:00 - 0:59h** | **1:00 - 1:59h** | **2:00 - 2:59h** | **3:00 - 3:59h** | **4:00 - 4:59h** | **5:00 - 5:59h** | **6:00 - 6:59h** | **7:00 - 7:59h** | **8:00 - 8:59h** | **9:00 - 9:59h** | **10:00 - 10:59h** | **11:00 - 11:59h** |
| lower 0.95 CI | 23.18 | 40.95 | 40.95 | 40.95 | 0.00 | 0.00 | 23.18 | 0.00 | 64.13 | 124.165 | 202.49 | 197.8 |
| median | 64.13 | 81.9 | 55.245 | 40.95 | 32.065 | 0.00 | 40.95 | 23.18 | 85.07 | 170.525 | 226.735 | 241.92 |
| upper 0.95 CI | 85.07 | 91.5 | 85.07 | 83.485 | 40.95 | 40.95 | 68.92 | 48.445 | 100.985 | 211.94 | 248.6 | 277.615 |
| Time (PM) | **12:00 - 12:59h** | **1:00 - 1:59h** | **2:00 - 2:59h** | **3:00 - 3:59h** | **4:00 - 4:59h** | **5:00 - 5:59h** | **6:00 - 6:59h** | **7:00 - 7:59h** | **8:00 - 8:59h** | **9:00 - 9:59h** | **10:00 - 10:59h** | **11:00 - 11:59h** |
| lower 0.95 CI | 212.865 | 188.76 | 156.85 | 149.2 | 133.05 | 191.27 | 146.03 | 128.26 | 107.785 | 85.07 | 64.13 | 85.07 |
| median | 254.01 | 223.335 | 209.29 | 207.92 | 188.565 | 205.465 | 170.795 | 165.385 | 146.03 | 126.02 | 96.195 | 122.85 |
| upper 0.95 CI | 295.23 | 257.835 | 243.81 | 242.69 | 233.805 | 227.2 | 214.45 | 193.975 | 170.795 | 163.8 | 115.28 | 147.81 |

| **Sunday** |  |  |  |  |  |  |  |  |  |  |  |  |
| --- | --- | --- | --- | --- | --- | --- | --- | --- | --- | --- | --- | --- |
| Time (AM) | **0:00 - 0:59h** | **1:00 - 1:59h** | **2:00 - 2:59h** | **3:00 - 3:59h** | **4:00 - 4:59h** | **5:00 - 5:59h** | **6:00 - 6:59h** | **7:00 - 7:59h** | **8:00 - 8:59h** | **9:00 - 9:59h** | **10:00 - 10:59h** | **11:00 - 11:59h** |
| lower 0.95 CI | 64.13 | 11.59 | 0.00 | 40.95 | 7.495 | 0.00 | 0.00 | 19.085 | 40.95 | 140.74 | 180.105 | 215.57 |
| median | 85.07 | 40.95 | 40.95 | 55.245 | 40.95 | 40.95 | 23.18 | 32.065 | 66.835 | 168.34 | 233.265 | 248.87 |
| upper 0.95 CI | 117.135 | 52.54 | 85.07 | 83.485 | 73.015 | 40.95 | 40.95 | 73.015 | 86.19 | 198.57 | 259.42 | 294.305 |
| Time (PM) | **12:00 - 12:59h** | **1:00 - 1:59h** | **2:00 - 2:59h** | **3:00 - 3:59h** | **4:00 - 4:59h** | **5:00 - 5:59h** | **6:00 - 6:59h** | **7:00 - 7:59h** | **8:00 - 8:59h** | **9:00 - 9:59h** | **10:00 - 10:59h** | **11:00 - 11:59h** |
| lower 0.95 CI | 251.575 | 219.045 | 178.1 | 150.32 | 151.44 | 146.03 | 124.63 | 127.14 | 85.07 | 83.485 | 81.9 | 81.9 |
| median | 294.535 | 276.34 | 204.95 | 183.6 | 175.085 | 168.09 | 129.845 | 169.21 | 120.59 | 115.55 | 87.31 | 108.25 |
| upper 0.95 CI | 325.21 | 312.535 | 247.13 | 207.65 | 233.805 | 191.27 | 168.09 | 188.76 | 166.97 | 131.43 | 108.25 | 126.02 |
|  |  |  |  |  |  |  |  |  |  |  |  |  |
| Confidence intervals were evaluated by non-parametric bootstrapping (10000 bootstrap samples per confidence interval) using SPSS 23 software (IBM SPSS, Chicago/IL. USA). | | | | | | | | | | | |  |
