# Supplementary material for: Nurse Staffing Calculation in the Emergency Department - Performance-Oriented Calculation Based on the Manchester Triage System at the University Hospital Bonn
Source: PLoS One. 2016 May 3;11(5):e0154344. doi: 10.1371/journal.pone.0154344 (PMC4854466; doi:10.1371/journal.pone.0154344)
Supplement: S5 Table — (DOCX) [file pone.0154344.s007.docx]

**S 5 Table. Staff shortfall times in the interdisciplinary emergency department***

| **2010** | | | | | | | |
| --- | --- | --- | --- | --- | --- | --- | --- |
|  | January | February | March | April | Mai | June |  |
| illness | 467,75 | 150,15 | 231 | 11,55 | 57,7 | 71,23 |  |
| holidays | 78,94 | 100,1 | 396,6 | 277,2 | 418,06 | 508,27 |  |
| special leave | 77 | 269,54 | 152,08 | 273,38 | 236,78 | 109,73 |  |
| total | 623,69 | 519,79 | 779,68 | 562,13 | 712,54 | 689,23 |  |
| gross working hours | 3799,96 | 3772,56 | 4338,97 | 3584,34 | 3584,34 | 3803,79 |  |
| % shortfall | 16,41 | 13,78 | 17,97 | 15,68 | 16,82 | 18,12 |  |
|  |  |  |  |  |  |  |  |
|  | July | August | September | October | November | December |  |
| illness | 51,98 | 415,86 | 196,35 | 236,79 | 302,28 | 471,77 |  |
| holidays | 614,1 | 883,58 | 581,36 | 546,7 | 424,9 | 388,85 |  |
| special leave | 84,7 | 96,36 | 354,24 | 269,5 | 315,7 | 396,56 |  |
| total | 750,78 | 1395,8 | 1131,95 | 1052,99 | 1042,88 | 1257,18 |  |
| gross working hours | 4204,21 | 4516,04 | 4319,3 | 4327 | 4487,19 | 4325,49 |  |
| % shortfall | 17,86 | 30,91 | 26,21 | 24,34 | 23,24 | 29,06 |  |
|  |  |  |  |  |  |  |  |

*mean of staff shortfall times and total gross working hours; Specified in hours per month / the individual shortfall rate calculated for the interdisciplinary emergency department is 20.87%.
